# Early feeding with hydrogel nutrients modifies cecal microbiota, immunity, gene expression, and improves growth performance in ostrich chicks

**DOI:** 10.1016/j.psj.2025.106025

**Published:** 2025-10-26

**Authors:** Mohamed marzok, Hind Althagafi, Hadeel A. Almamoory, AbdelRahman Y. Abdelhady, Mohamed G. Sallam, Mahmoud H.A. Mohamed, Mohammed Al-Rasheed, Ahmed Ateya, Salah Abdulaziz Al-Shami, Ahmed I. El Sheikh, Sherief M. Abdel-Raheem, Moustafa Salouci, Khairiah Mubarak Alwutayd, Ahmed M. Elbaz

**Affiliations:** a Department of Clinical Sciences, College of Veterinary Medicine, King Faisal University, P.O. Box: 400, Al-Ahsa 31982, Saudi Arabia; b Department of Biology, College of Science, Princess Nourah bint Abdulrahman University, P.O. Box 84428, Riyadh 11671, Saudi Arabia; c Department of Animal Production Techniques, Technical College of Al Musaib, Al Furat Al Awsat Technical University, Babylon, Iraq; d Poultry Production Department, Faculty of Agriculture, Ain Shams University, Cairo, Egypt; e Animal Production Department, Agricultural and Biology Research Institute, National Research Centre, Cairo, Egypt; f Department of Development of Animal Wealth, Faculty of Veterinary Medicine, Mansoura University, Mansoura, Egypt; g Department of Public Health, College of Veterinary Medicine, King Faisal University, P.O. Box 400, Al-Hofuf 31982, Al-Ahsa, Saudi Arabia; h Anatomy department, college of Veterinary Medicine. King Faisal University, Saudi Arabia; i Animal and Poultry Nutrition Department, Desert Research Center, Mataria, Cairo, Egypt

**Keywords:** Ostrich performance, Early feeding, Immunity, Intestinal microbiota, Gene expression

## Abstract

The period extending from the final days of embryonic development until post-hatching is critical for the development of the digestive and immune systems of poultry. Therefore, early feeding programs are used to provide all the nutrients and growth factors necessary to support the maturation and integrity of the digestive system. Accordingly, this study aimed to evaluate the effect of early post-hatch feeding with hydrated nutrient gel (HNG) on growth performance, immune response, cecal microbiota, and gene expression in ostrich chicks. One hundred and fifty African ostrich chicks were divided immediately after hatching into six groups (five replicates per group) as follows: the first group was feed deprived for the first 24 h after hatching (control group, HNG0); the other groups was feed deprived for the first 2 (HNG2), 6 (HNG6), 12 (HNG12), 18 (HNG18), and 24 (HNG24) hours post-hatching, respectively (given 25 g HNG/chick). Results showed that receiving HNG2 and HNG6 groups significantly increased the body weight gain (BWG); however, the feed conversion ratio (FCR) decreased (*P < 0.05*). Chicks fed HNG2 and HNG6 had higher (*P < 0.05*) superoxide dismutase (SOD), immunoglobulin A (IgA), triiodothyronine (T3), and interleukin-10 (IL-10) contents than those fed other treated-groups and HNG0, whereas lower malondialdehyde (MDA) and interleukin-6 (IL-6) levels (*P < 0.05*). In the HNG2 and HNG6 groups, the *Escherichia coli* count significantly decreased, while the *Lactobacillus* count increased in chicks fed HNG2, HNG6, and HNG12 (*P < 0.05*). Additionally, IGF-1 gene expression was significantly increased in HNG2 and HNG6 groups (*P < 0.05*). In summary, early feeding with HNG can effectively improve growth performance, antioxidant capacity, immunity, cecal microbiota, and gene expression of ostrich chicks. This study presents an effective strategy for applying early HNG feeding in ostrich chicks.

## Introduction

The most critical stage of a chicken's life is the first few hours after hatching, during which radical functional developments occur in various body systems, including the immune and digestive systems (Hesabi Nameghi et al., 2022). This stage is influenced by numerous internal and external factors, including genetic traits, nutrition, and the surrounding conditions before and after laying, which play a major role in the bird's performance and survival (Gaweł et al., 2022; Yerpes et al., 2020 ). Variations in hatching time and chick placement in the rearing area also lead to a range of stressful events, including early deprivation of food and water, transportation, dehydration, and pollution (Jha et al., 2019; Hesabi Nameghi et al., 2022). These events make them vulnerable to impaired immune, muscular, and digestive system development, leading to poor performance, poor health, and increased mortality (Hedlund et al., 2019). Especially since many researchers have reported that the immune and digestive systems are immature (not fully developed and functional) in newly hatched chicks (Gaweł et al., 2022; Ravindran et al., 2021). It has been proven that delayed access to water and feed in newly hatched chicks negatively affects intestinal morphology, immune response, gut microbiota, and benefits from nutrients, leading to dehydration, energy depletion, and, consequently, deterioration in broiler performance (Li et al., 2022). Metabolic changes caused by delayed access to feed in newborn chicks also lead to increased susceptibility to disease, reduced lymphoid organ development, and a weakened immune response (Shinde et al., 2015). Therefore, early feeding may have an effective impact on improving yolk utilization and enhancing the development of the immune and digestive systems. In addition, early feeding of easily digestible nutrients instead of yolk sac nutrients may accelerate the growth and adaptation of the digestive system and support immune response and metabolic pathways, improving feed efficiency and promoting the health of young chicks (Hesabi Nameghi et al., 2022). For this reason, several early feeding systems have been developed, including in-egg feeding systems and hatchery or cage feeding systems for chicks during transport (Abdel-Moneim et al., 2020; da Silva et al., 2021).

Recently, ostrich (*Struthio camelus*) farms have spread across North Africa, mainly because the climate is suitable for raising ostriches and because this large desert bird can withstand various environmental conditions (da Silva et al., 2021). However, there are gaps in knowledge regarding the early post-hatching stages in ostrich farming. Studies on ostriches are limited, with most focusing on broiler chickens, which represent the primary source of poultry protein worldwide. Ostrich farms suffer from high mortality rates during the early post-hatching stages. Some breeders, through veterinarians supervising the farms, attributed this to digestive disorders (Kivi et al., 2015). Some have recommended not feeding ostrich chicks within 24 to 36 h of hatching; however, the mortality rate has not significantly decreased. Several researchers have reported that depriving broiler chickens of feed and water during the first hours after hatching has long-term negative effects on their growth and survival (Li et al., 2022; Hesabi Nameghi et al., 2022). Accordingly, the health and nutritional status of chicks during the early stages of hatching are of great importance for their health throughout their life cycle (Gaweł et al., 2022). Additionally, several studies have been conducted on early feeding of ostrich chicks through in-ovo injection of nutrients, which have proven that embryonic nutrition enhances growth performance and gut microbes (Elbaz et al., 2025a,b). Despite that, we did not reach the desired results regarding reducing the mortality rate and enhancing growth performance. Therefore, we considered studying early feeding during the first hours after hatching, which has proven successful in many studies on broiler chickens (Hollemans et al., 2018; Jha et al., 2019).

Therefore, providing hydrated nutritious gel during the first hatch period of ostrich chicks may be an effective and easy-to-implement method for providing a wide range of easily digestible, highly effective nutrients at one day of age, thereby promoting the growth and development of the digestive system, skeletal muscles, and immunity. Thence, this study aimed to evaluate the effect of early feeding of HNG during different post-hatch times on growth, antioxidant status, immune response, cecal microbiota, and gene expression in ostrich chicks.

## Methods and materials

### Ethics statement

The experiment followed the animal safety, welfare, and ethics guidelines of the Faculty of Agriculture, Ain Shams University, and Desert Research Center, Cairo, Egypt (DRC, 0018-024-164).

### Experimental design and diets

Fertilized ostrich eggs were purchased from a commercial farm in Alexandria, Egypt. Eggs were transferred and incubated in a commercial hatchery (HEKA-Ostrich-Incubator "Steppe", by European Middle Eastern Group for Technology, Consultancy and Trade (EMEG4TCT), Egypt) at the research station of the Desert Research Center. One hundred and fifty African ostrich chicks were divided immediately after hatching into six groups (five replicates per group) as follows: HNG0 was deprived of feed for the first 24 h after hatching (control group); HNG2 was deprived of feed for the first 2 h after hatching and then received 25 g HNG/ chick; HNG6 was deprived of feed for the first 6 h after hatching and then received 25 g HNG/ chick; HNG12 was deprived of feed during the first 12 h of hatching and then received 25 g HNG/ chick; HNG18 was deprived of feed during the first 18 h of hatching and then received 25 g HNG/ chick; and HNG24 was deprived of feed during the first 24 h of hatching and then received 25 g HNG/ chick. The experiment lasted for 12 weeks. A previous study, currently being published, was conducted to determine the optimal amount of HNG supplementation, which was 25 g per chick, then access to regular feed. After distributing the chicks to replicates, the HNG was divided into 25-gram weights and placed in front of each bird individually according to the time allotted for each group. Commercial feed was obtained from the farm from which the chicks were purchased, and its composition is shown in Table 1. After completing the experimental treatments within the first 24 h of hatching, all groups were fed a crumble starter feed until the end of the experimental period, with access to water immediately after hatching. Chicks are housed in separate replicates (5 chicks) within each group (5 replicates) on a rough concrete floor with continuous cleaning to maintain the chicks’ safety, and provided with adequate husbandry conditions with continuous monitoring of ventilation. The feed was analyzed to determine its nutrient content in the laboratories of the Desert Research Center according to the AOAC (2000) method. AVIBOOST (Aqua-Blok Reg. No. V23082 (Act 36/1947)) was purchased from the International Free Trade Company in Egypt, and produced by Ashkan Consulting (Pty) Ltd., South Africa. The nutritional gel used in this study, AVIBOOST, contains some vitamins (A, D3, E, B1, B2, B6, B12, C, K3, and biotin), organic and amino acids (folic acid, pantothenic acid, citric acid, lysine, and methionine), trace elements (manganese, copper, selenium, iodine, and zinc), electrolytes (potassium, sodium, and cobalt), and essential fatty acids. Supplement file

**Table 1:** Composition and chemical analysis of the basal diet of ostrich chicks.

**Ingredient**	**Starter (0 to 8 wk)**	**Grower (8-12 wk)**
Yellow Corn	48.7	56.6
Soybean meal (44 %)	27.1	21.7
Sunflower Meal (40 %)	3.50	3.00
Alfalfa meal	3.80	3.80
Corn Gluten meal (62 %)	2.00	2.00
Wheat bran	6.00	6.00
Soybean Oil	3.00	1.00
Calcium Carbonate	1.57	1.53
Monocalcium phosphate	2.55	2.55
Premix*	1.00	1.00
NaCl	0.30	0.30
DL-Methionine	0.25	0.25
HCL Lysine	0.13	0.17
Sodium Bicarbonate	0.10	0.10
**Chemical analysis**		
ME (Kcal/kg)	2786	2721
Crude protein	20.09	18.13
Calcium	1.171	1.139
Available Phosphorus	0.629	0.653
Lysine	1.125	1.023
Methionine	0.581	0.552

⁎ Premix, each 1 kg consists of: Vit. A, 13,000 I.U; Vit. D3, 4,500 I.U; Vit. E, 21 IU; D3, 4,500 I.U; Vit. K3, 3.7 mg; Vit. B2, 8 mg; Vit. B1, 4 mg; Vit. B12, 17 mg; Vit. B6, 5 mg; Niacin, 60 mg; D-Biotin, 200 mg; Folic acid, 2.1 mg; Calcium, 16.18 mg; manganese, 100.0 mg; iron, 80.0 mg; ZnSO4H2O, 200 mg; CuSO4H2O, 31.18 mg; selenium, 250.0 mg; iodine, 2.0 mg; and cobalt, 500.0 mg.

### Growth performance

Chicks were weighed individually immediately after hatching and randomly distributed among experimental groups. Live body weight (LBW, g) of individual chicks and feed intake (FI, g) were recorded at 4, 8, and 12 weeks of age, as well as body weight gain (BWG) and feed conversion ratio (FCR, FI (g)/ BWG (g)) was calculated (adjusted for mortality). Daily mortality was recorded and the mortality rate was calculated at the end of the experimental period.

### Plasma biochemistry

At week 12, blood samples were drawn from the jugular vein of 30 chicks (five chicks/group). To obtain plasma, blood was placed in anticoagulant tubes, centrifuged at 3,400 × g for 9 min, and stored at -20 °C. Plasma levels of immunoglobulin A (IgA), immunoglobulin M (IgM), and immunoglobulin G (IgG) were quantified using chicken-specific ELISA kits (Life Diagnostics Inc., PA, USA). Plasma concentrations of interleukin 10 (IL-10) and interleukin 6 (IL-6) were analyzed using commercially available ELISA kits according to the manufacturer's protocols (MyBioSource, San Diego, CA). Additionally, the activity of superoxide dismutase (SOD) and glutathione peroxidase (GPx) enzymes and the level of malondialdehyde (MDA) in plasma were determined using commercial analysis kits (Spinreact Co. Girona, Spain). A corticosterone ELISA kit was obtained from Cayman Chemical Company in Ann Arbor, Michigan, in the United States, to measure plasma corticosterone (COR). Analysis of blood triiodothyronine (T3) concentrations was performed by using commercial chicken ELISA kits (MyBioSource, Inc., San Diego, CA).

### Cecal microbiota

During slaughter, from five chicks per experimental group, 10 g of digesta cecum sample/chick were weighed into sterile bags for bacteriological examinations. Appropriate serial dilutions of the experimental samples were performed. A portion of the diluted samples was transferred using a 1 ml fixed pipette to Petri dishes for inoculation into different nutrients and selective media specific to each target microbe. Microbial assays included *Lactobacillus, Clostridium perfringens* (*C. perfringens*), and *Escherichia coli* (*E. coli*), according to Czerwiński et al. (2012) and Elbaz et al. (2021). De Man Rogosa Sharpe (MRS agar, Merck, Darmstadt, Germany) agar medium was prepared for *Lactobacillus* spp. growth and incubated at 37 °C for 48 h under anaerobic conditions. Sulfite Indole Motility (SIA agar, Merck, Darmstadt, Germany) agar medium was prepared for *C. perfringens* growth and incubated at 35 °C for 24 h. MacConkey agar medium was prepared for *E. coli* growth (as lactose-fermenting bacteria) and incubated at 35 °C for 72 h.

### Gene expression

Five ostriches from each group were slaughtered, and samples of their mid-small intestine (jejunum) and liver were obtained right away. MUC2 was expressed in the small intestine, and IGF-1 in liver tissue. Total RNA was extracted using the Trizol reagent (Tiangen Bio-tech Co., Ltd., Beijing, China) in accordance with the manufacturer's instructions. Complementary RNA was synthesized using the PrimeScript RT reagent kit (Takara Biotechnology Co. Ltd., Beijing, China). Using the SYBR Premix Ex Taq diagnostic kit (Takara Biotechnology Co. Ltd., Beijing, China), quantitative real-time PCR (qRT-PCR) reactions were created. The gene and its related primers are described as follows: IGF-1 forward: 5′- GCCATCTGCAGGATACTTTGC-3′, reverse: 5′- CTGGGAGAATGCCCATTGGT-3′ (Accession No., AB035804.1) with expected amplicon size 114-bp, and MUC2 forward: 5′- CCACAGTGCTCTTCAGTCGT-3, reverse: 5′- TGGCAGCATAGACCTGCAAA-3′ (Accession No., XM_068944759.1) with expected amplicon size 220-bp.

The ß. actin gene's primer sequence was as follows: 5′- CATCACAAGGGTGTGGGTGT-3′ in reverse and 5′- GCG-CAAGTACTCTGTCTGGA-3′ in forward (Acces-sion No., KJ729106.1). After putting the finished reaction mixture in a thermal cycler, the following steps were performed: reverse transcription for 30 min at 50 °C, primary denaturation for 10 min at 94 °C, 40 cycles of secondary denaturation for 15 s at 94 °C, annealing temperatures for 15 s at 58 °C, and extension temperature for 30 s at 72 °C. The 2−ΔΔCt was used to calculate the relative expression of the MUC2 and IGF-1 genes per sample in comparison to the ß. actin gene (Livak and Schmittgen, 2001).

### Statistical analysis

Using SPSS statistical software (version 19.0 for Windows; SPSS Inc., Chicago, IL), experimental data were analyzed using one-way analysis of variance (ANOVA). Tukey’s multiple comparison test. Results were expressed as mean and ± standard deviation (± SD), and differences were considered significant at *P < 0.05*.

## Results

### Growth performance

**Fig. 1:**
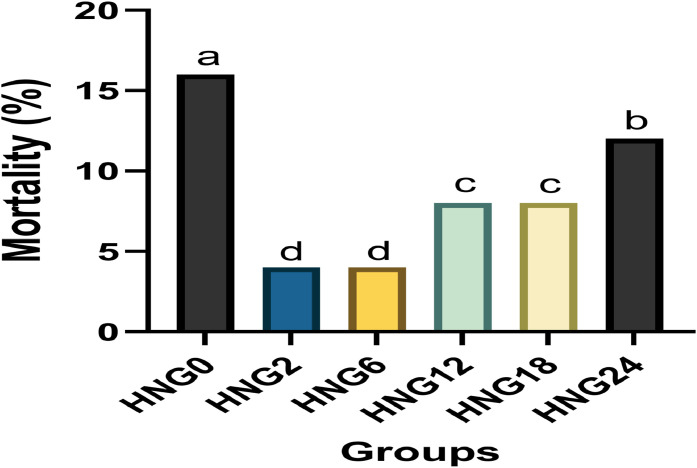
Effect of early feeding on mortality rate (%) in ostrich chicks. HNG0 was fed deprived for the first 24 h after hatching (control group); HNG2 was fed deprived for the first 2 h after hatching and received 25 g HNG/ chick; HNG6 was fed deprived for the first 6 h after hatching and received 25 g HNG/ chick; HNG12 was fed deprived during the first 12 h of hatching and received 25 g HNG/ chick; HNG18 was fed deprived during the first 18 h of hatching and received 25 g HNG/ chick; and HNG24 was fed deprived during the first 24 h of hatching and received 25 g HNG/ chick. a-d Means within a row lacking a common superscript differ significantly (P < 0.05).

Compared with the HNG0 group, the BWG of ostrich chicks was significantly increased in HNG2, HNG6, and HNG12 groups at 4 weeks (*P < 0.05*; Table 2). Moreover, the HNG2, HNG6, HNG12, and HNG18 groups had higher BWG (*P < 0.05*) than the other groups at 8 and 12 weeks (*P < 0.05*). However, the best BWG was in the HNG2 and HNG6 groups. Moreover, all groups receiving HNG showed a lower FCR than the control group during all experimental periods (*P < 0.05*); while the best FCR was in the HNG2 and HNG6 groups (*P < 0.05*). However, there were no differences in FI among experimental groups during the different periods (*P < 0.05*). Additionally, mortality rate was significantly reduced in HNG2 and HNG6 groups during the overall period, compared to the other groups (P = 0.016, Fig. 1).

**Table 2:** Effects of early feeding on growth performance in ostrich chicks at 4, 8, and 12 weeks.

Items	HNG0				HNG2	HNG6	HNG12	HNG18	HNG24	*P*-Value
**IBW, g**	927 ± 11.3				921 ± 12.0	930 ± 9.28	926 ± 8.74	932 ± 10.3	923 ± 9.57	0.147
**Body weight gain, g**										
4 wk	3067 ± 17.8^c^				3212 ± 19.3^a^	3209 ± 18.6^a^	3158 ± 18.8^b^	3127 ± 19.7^bc^	3104 ± 21.0^bc^	0.001
8 wk	9081 ± 24.1^e^				9615 ± 23.4^a^	9597 ± 20.3^a^	9342 ± 22.1^b^	9263 ± 23.8^c^	9145 ± 21.7^de^	<0.001
12 wk	13754 ± 25.3^e^				14528 ± 27.8^a^	14503 ± 27.6^a^	14406 ± 28.6^b^	14288 ± 29.4^c^	14019 ± 26.5^d^	<0.001
**Feed intake, g**										
4 wk	5156 ± 15.3				5149 ± 13.8	5151 ± 15.7	5143 ± 16.9	5154 ± 17.3	5158 ± 15.8	0.218
8 wk	16532 ± 25.1				16551 ± 23.6	16543 ± 24.1	16535 ± 22.4	16544 ± 21.8	16536 ± 22.6	0.503
12 wk	29065 ± 30.4				29077 ± 28.2	29081 ± 29.7	29072 ± 32.4	29058 ± 29.3	29067 ± 33.0	0.244
**Feed conversion ratio, g/g**										
4 wk	1.681 ± 0.02^a^				1.603 ± 0.04^d^	1.605 ± 0.06^d^	1.630 ± 0.05^c^	1.649 ± 0.03^bc^	1.663 ± 0.04^b^	<0.001
8 wk	1.821 ± 0.00^a^				1.722 ± 0.00^e^	1.724 ± 0.01^e^	1.770 ± 0.03^d^	1.786 ± 0.00^cd^	1.809 ± 0.01^b^	<0.001
12 wk	2.113 ± 0.03^a^				2.001 ± 0.01^e^	2.005 ± 0.00^e^	2.018 ± 0.01^dc^	2.034 ± 0.00^c^	2.074 ± 0.01^b^	<0.001

a-e Means within a row lacking a common superscript differ significantly (*P* < 0.05); ± SD - standard deviation. HNG0 was fed deprived for the first 24 h after hatching (control group); HNG2 was fed deprived for the first 2 h after hatching and received 25 g HNG/ chick; HNG6 was fed deprived for the first 6 h after hatching and received 25 g HNG/ chick; HNG12 was fed deprived during the first 12 h of hatching and received 25 g HNG/ chick; HNG18 was fed deprived during the first 18 h of hatching and received 25 g HNG/ chick; and HNG24 was fed deprived during the first 24 h of hatching and received 25 g HNG/ chick.

### Stress and antioxidative markers

**Fig. 2:**
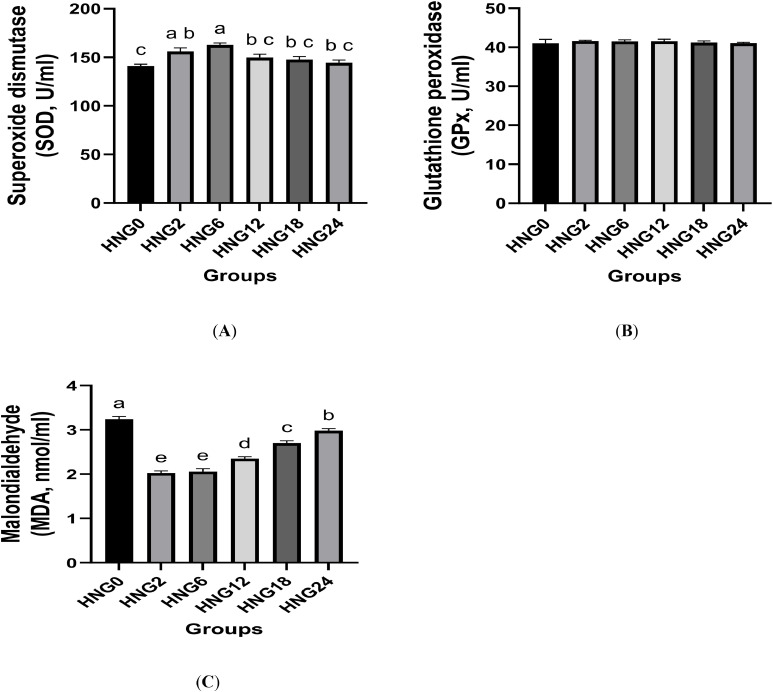
Effect of early feeding on superoxide dismutase (SOD, A), glutathione peroxidase (GPx, B), and malondialdehyde (MDA, C) in ostrich chicks at 12 weeks. HNG0 was fed deprived for the first 24 h after hatching (control group); HNG2 was fed deprived for the first 2 h after hatching and received 25 g HNG/ chick; HNG6 was fed deprived for the first 6 h after hatching and received 25 g HNG/ chick; HNG12 was fed deprived during the first 12 h of hatching and received 25 g HNG/ chick; HNG18 was fed deprived during the first 18 h of hatching and received 25 g HNG/ chick; and HNG24 was fed deprived during the first 24 h of hatching and received 25 g HNG/ chick. SOD, superoxide dismutase; GPx, glutathione peroxidase; MDA, malondialdehyde. a-e Means within a row lacking a common superscript differ significantly (P < 0.05). All data are expressed as the mean ± SD.

Early feeding affected stress markers as shown in Table 3. Levels of T3 increased (*P < 0.05*) in ostrich chicks in HNG2 and HNG6 groups compared to the other groups, while COR levels were not affected (Table 3). Additionally, early feeding enhanced antioxidant ability by decreasing MDA levels and increasing SOD activity in the HNG2 and HNG6 groups (*P < 0.05*) compared to the other groups (Fig. 2, (A, B, and C)), while GPx activity was unaffected.

**Table 3:** Effects of early feeding on stress markers and immune response (immunoglobulin and immune cytokines) in ostrich chicks at 12 weeks.

Items	HNG0	HNG2	HNG6	HNG12	HNG18	HNG24	*P*-Value
COR, ng/ml	16.3 ± 1.29	15.9 ± 1.32	15.8 ± 1.36	16.2 ± 1.06	16.1 ± 1.22	16.2 ± 1.41	0.204
T3, ng/ml	0.74 ± 0.02^b^	0.85 ± 0.03^a^	0.86 ± 0.01^a^	0.79 ± 0.02^ab^	0.75 ± 0.01^b^	0.74 ± 0.02^b^	0.001
IgG, mg/dl	622 ± 6.94	619 ± 7.55	631 ± 9.12	624 ± 8.64	627 ± 10.2	617 ± 9.65	0.157
IgM, mg/dl	143 ± 0.93	152 ± 0.84	155 ± 0.85	148 ± 0.78	150 ± 1.21	146 ± 1.03	0.072
IgA, mg/dl	261 ± 2.75^b^	294 ± 2.92^a^	288 ± 3.14^a^	272 ± 3.38^ab^	263 ± 2.65^b^	259 ± 3.02^b^	0.001
IL-10, pg/dl	41.3 ± 0.20^c^	53.4 ± 0.16^a^	55.8 ± 0.30^a^	47.3 ± 0.19^b^	44.9 ± 0.22^bc^	43.6 ± 0.16^bc^	0.001
IL-6, pg/dl	92.5 ± 1.01^a^	89.7 ± 1.09^b^	90.3 ± 1.05^b^	91.2 ± 1.03^ab^	92.0 ± 0.78^a^	92.3 ± 0.91^a^	0.040

a-c Means within a row lacking a common superscript differ significantly (*P* < 0.05); ± SD - standard deviation. HNG0 was fed deprived for the first 24 h after hatching (control group); HNG2 was fed deprived for the first 2 h after hatching and received 25 g HNG/ chick; HNG6 was fed deprived for the first 6 h after hatching and received 25 g HNG/ chick; HNG12 was fed deprived during the first 12 h of hatching and received 25 g HNG/ chick; HNG18 was fed deprived during the first 18 h of hatching and received 25 g HNG/ chick; and HNG24 was fed deprived during the first 24 h of hatching and received 25 g HNG/ chick. COR, Corticosterone; T3, triiodothyronine; IgA, immunoglobulin A; IgM, immunoglobulin M; IgG, immunoglobulin G; IL-10, interleukin 10; IL-6, interleukin 6.

### Immune response

Early feeding demonstrated a modulatory effect on the immune response of ostrich chicks, as shown in Table 3. Blood levels of IgA increased (*P < 0.05*) in the HNG2 and HNG6 groups compared to the other groups, while levels of IgM and IgG were unaffected by the experimental treatments (*P < 0.05*). Similarly, levels of IL-10 increased and levels of IL-6 decreased in the HNG2 and HNG6 groups compared to the other groups (*P < 0.05*). Despite that, there was a numerical increase in IgM levels in the HNG2 and HNG6 groups (P = 0.072).

### Cecal microbiota

Table 4 shows the effect of early feeding on the gut microbiome diversity in ostrich chicks. Early feeding had a modulatory effect on the gut microbiome, increasing *Lactobacillus* counts and decreasing *E. coli* counts (*P < 0.05*) in HNG2 and HNG6 groups compared to the other groups; however, *C. perfringens* counts were unaffected (*P < 0.05*).

**Table 4:** Effects of early feeding on intestinal microbial community (log10 CFU g^−1^) in ostrich chicks at 12 weeks.

Items	HNG0	HNG2	HNG6	HNG12	HNG18	HNG24	P-Value
*Lactobacillus*	5.3 ± 0.31^c^	6.9 ± 0.28^ab^	7.8 ± 0.27^a^	6.2 ± 0.30^b^	5.7 ± 0.24^bc^	5.2 ± 0.31^c^	0.001
*C. perfringens*	1.74 ± 0.07	1.68 ± 0.03	1.71 ± 0.04	1.79 ± 0.04	1.75 ± 0.06	1.72 ± 0.04	0.146
*E. coli*	3.5 ± 1.03^a^	2.7 ± 0.94^b^	2.3 ± 1.01^b^	3.1 ± 1.12^ab^	3.0 ± 0.87^ab^	3.4 ± 0.96^a^	0.020

a-c Means within a row lacking a common superscript differ significantly (*P* < 0.05); ± SD - standard deviation. HNG0 was fed deprived for the first 24 h after hatching (control group); HNG2 was fed deprived for the first 2 h after hatching and received 25 g HNG/ chick; HNG6 was fed deprived for the first 6 h after hatching and received 25 g HNG/ chick; HNG12 was fed deprived during the first 12 h of hatching and received 25 g HNG/ chick; HNG18 was fed deprived during the first 18 h of hatching and received 25 g HNG/ chick; and HNG24 was fed deprived during the first 24 h of hatching and received 25 g HNG/ chick. *C. perfringens, Clostridium perfringens,* and *E. coli, Escherichia coli*.

### Gene expression

**Fig. 3:**
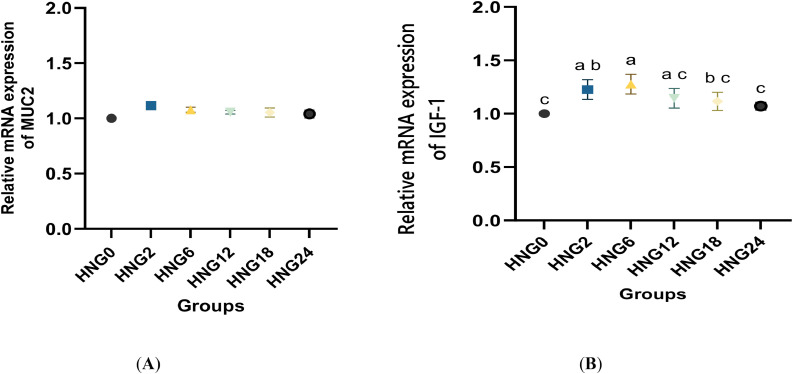
Effect of early feeding on the mRNA expression of mucin 2 (MUC2, A) in the small intestine and Insulin-like Growth Factor 1 (IGF-1, B) in liver tissue of ostrich chicks at 12 weeks. HNG0 was fed deprived for the first 24 h after hatching (control group); HNG2 was fed deprived for the first 2 h after hatching and received 25 g HNG/ chick; HNG6 was fed deprived for the first 6 h after hatching and received 25 g HNG/ chick; HNG12 was fed deprived during the first 12 h of hatching and received 25 g HNG/ chick; HNG18 was fed deprived during the first 18 h of hatching and received 25 g HNG/ chick; and HNG24 was fed deprived during the first 24 h of hatching and received 25 g HNG/ chick. a-e Means within a row lacking a common superscript differ significantly (P < 0.05). All data are expressed as the mean ± SD.

In Fig. 3 (A and B), the IGF-1 gene expression significantly increased (*P < 0.05*) in the HNG2 and HNG6 groups (*P < 0.05*) compared to the other groups, while expression of the MUC2 gene was not affected. However, the highest gene expression was in the HNG6 group than in the other groups (*P < 0.05*). Despite that, there was a numerical increase in expression of the MUC2 gene in the HNG2, HNG6, and HNG12 groups (P = 0.061).

## Discussion

Early feeding is a modern nutritional strategy used to support the health and performance of newborn chicks by adding essential nutrients before the digestive system matures (Jha et al., 2019). Therefore, this study examined the potential for supporting the performance of newborn ostrich chicks through early feeding of HNG.

The results of the current study showed that ostrich chicks that did not receive hydrated nutrient gel had lower weight gain than those that received hydrated nutrient gel. The results indicate that depriving chicks of a nutritional source after hatching has a negative impact on performance; however, early feeding with hydrated nutrient gel mitigated the negative impacts of early nutritional stress. In this context, Willemsen et al. (2010) found that early fasting of newborn chicks led to decreased growth performance. Similarly, Gaweł et al. (2022) reported that feed deprivation reduced the rate of yolk sac absorption and growth performance during the first days after hatching. Additionally, when the initiation of external feeding after hatching is delayed, the growth and development of the chicks' digestive and immune systems are impaired (Jha et al., 2019), which in turn, may have a detrimental effect on greater susceptibility to diseases and feed efficiency. Notably, our results showed that early feeding of HNG significantly improved body weight and feed conversion ratio through the experimental period, especially during the first hours of hatching (HNG2 and HNG6). Consistent with our results, several previous reports have shown that early feeding significantly increased total body weight and decreased total feed conversion ratio (Gaweł et al., 2022; Hesabi Nameghi et al., 2022). The improved growth performance of newly hatched chicks fed early-stage feeding may be due to the provision of certain essential nutrients necessary for chicks to overcome nutrient deficiencies during late incubation, hatching challenges, environmental conditions, and disease challenges, as well as supporting their adaptation to changing nutritional conditions and digestive and immune system growth and development (Yadav and Jha, 2019). Additionally, using early feeding from a feed (such as hydroponic nutrient gel) containing a higher concentration of digestible nutrients prepares the young chicks to digest the complex nutrients in the diet later, once the digestive system is mature (Jha et al., 2019), which improves the productive performance of chicks. Moreover, previous reports indicate that early feeding leads to faster consumption of fat-rich yolks as a food source, which stimulates physiological changes, functional development, and maturation of the digestive system (Lamot et al., 2014), including villus length and surface area (Sözcü et al., 2020), which supports growth performance. Interestingly, several reports have demonstrated the role of early feeding in modifying the expression of several genes that promote muscle growth, such as MFR4 and myoG, which promote the differentiation of growth-stimulating satellite cells (Peebles, 2018). Early feeding within the first few hours after hatching has been reported to have a positive effect on body weight, which increased by 4–6 % in the first week of life (Lingens et al., 2021). Additionally, in this study, the HNG2 and HNG6 groups had significantly lower mortality rates compared to the other groups. Lower mortality rates in chicks receiving HNG reflect improved physiological performance, immune response, and gut health. From the above, early feeding can have a positive impact on the growth performance of ostrich chicks through early stimulation of the digestive system and immunity.

During the period that chicks spend in the hatching chamber until they receive water and feed, the bird is exposed to several stressors, including hatchery conditions and drought, which impair their physiological growth (Hedlund et al., 2019; Varun et al., 2021). Stress markers assessed include changes in COR and T3 levels (Rajkumar et al., 2015). The results of the current study showed no change in COR levels, whereas decreased T3 levels were observed as a result of feed deprivation. This indicates that the activity of the hypothalamic-pituitary-adrenal axis, the primary producer of COR, is not affected by the duration of incubation, measured in hours (Lamot et al., 2014; Rajkumar et al., 2015), which is consistent with our study. Despite that, several reports indicate that stress significantly affects thyroid activity, as evidenced by lowered T3 levels in chicks, which is part of a broader stress response (Rajkumar et al., 2015). Our results show lower T3 levels in ostrich chicks exposed to food deprivation compared to other groups, as well as T3 levels increased with early feeding. This decreased thyroid hormone production may be a mechanism for reducing metabolic heat production during stress, which may help birds adapt to stressful conditions (Abdel-Moneim et al., 2021), especially since thyroid hormone plays an important role in many physiological processes, most importantly regulating metabolism. Consistent with our findings, several reports have demonstrated the role of feed supplements in enhancing thyroid function by increasing thyroid hormone production (El-Baz and Khidr, 2024; Madej et al., 2024), which supports physiological performance, growth, and overall health of chicks during stress. Similarly, Sözcü et al. (2020) found elevated plasma levels of T3 and T4 hormones in chicks fed FA early. From the above, early feeding can have an effective impact in mitigating the effects of stress in newly hatched ostrich chicks.

Some immune responses were examined in the current study because the immune system does not function at its full capacity and effectiveness in the post-hatching period (Jha et al., 2019). Providing several nutrients is important for promoting early immune system development. These include vitamin A, selenium, linoleic acid, and some B vitamins, especially during the late brooding and early post-hatching period in chicks (Prabakar et al., 2016), when much of the immune tissue growth occurs. Therefore, early feeding plays a significant role in modulating the immune system (Jha et al., 2019). The results of the current study showed a positive effect of early feeding on the immune response of newborn ostrich chicks, as it increased the levels of IgA and IL-10 and decreased the levels of IL-6. Consistent with our findings, early feeding enhances the immune response through several effects, including increased antibody production (Bakyaraj et al., 2012), immunoglobulin production, anti-inflammatory cytokine production, and increased organ weights (Kadam et al., 2013). Early feeding may play a role in stimulating immunity by providing the necessary micronutrients and substrates during the first days after hatching, supporting the proliferation of gut-associated lymphoid cells (Jha et al., 2019), which an essential for strengthening the immune system of chickens. Other mechanisms have also been proposed to explain the effects of early feeding on enhancing immune development, these mechanisms include: promoting faster yolk sac absorption immediately after hatching, which is essential for the absorption of maternal immunoglobulins and nutrients (Surai et al., 2016), thus supporting the immune system of newborn chicks, enhancing disease resistance, and improving overall health and performance.

Period from hatching until access to feed, water is a critical stage; chicks are more susceptible to oxidative damage (Kishawy et al., 2024), which leads to cell and tissue damage, resulting in weakened immunity, increased susceptibility to disease, and slowed growth. Providing chicks with immediate access to feed and water after hatching is an effective way to support their antioxidant defense systems (Jha et al., 2019; Riva et al., 2020), which positively impacts chick vitality and health. This is consistent with the results of our study, where early feeding led to increased SOD activity and decreased MDA levels. Antioxidant enzymes, including SOD, can mitigate elevated peroxide products, supporting the ability to maintain antioxidant and peroxide balance (Riva et al., 2020). Antioxidant capacity can also be improved by promoting gut microbiome composition through various factors, including modulating immune responses, producing antioxidant compounds, and regulating oxidative stress pathways (Kishawy et al., 2024; Elbaz et al., 2025a). The HNG used in the current study also contains a percentage of selenium, which allows for the formation of selenium reserves in tissues, especially muscles, and enhances antioxidant defenses (Berrocoso et al., 2017, Ron et al., 2020). Early feeding can play a crucial role in supporting the development of strong antioxidant defenses in chickens, leading to improved health, growth, and productive performance.

Understanding and enhancing gut health through various nutritional strategies is important in the poultry industry, especially during the early stages of hatching, as gut health is linked to nutrient utilization efficiency, immune system status, and microbiota stability (Li et al., 2022). The first period after hatching is also the period of rapid growth of the new chicks and high metabolism rate, which may lead to the depletion or deficiency of many essential nutrients (Abou-Elnaga et al., 2018; Jha et al., 2019), exposing the chicks to the challenges of diseases and the poor development of the digestive system. Numerous studies have demonstrated the importance of early nutrition and its impact on gut health by enhancing the immune system, intestinal weight, histological morphology, and microbiota (Hollemans et al., 2018; Li et al., 2022; Marcato et al., 2024). In our study, early feeding enhanced the gut microbiome by increasing *Lactobacillus* and decreasing *E. coli*. Previous studies reported that chicks fed directly post-hatch had increased body weight, which was attributed to mechanical stimulation of gut growth and development (Givisiez et al., 2020). This may be due to the positive effect of early feeding on the gut microbiota (Waghmare et al., 2025), through the effects of different post-hatching nutrients on the gut microbiota and tissue morphology. Additionally, hydrated nutrient gel contains some organic acids, which reduce the growth of harmful bacteria by lowering the environmental pH in the gut, making it less suitable for the proliferation of harmful bacteria. Furthermore, Furthermore, they can elicit non-pH direct toxic effects on bacterial metabolism, and can also prevent pathogen livability on the cellular level through their ability to target the cell wall, cytoplasmic membrane, and specific functions in cytoplasmic metabolism (Dittoe et al., 2018), as well as disrupting nutrient transport systems to pathogenic bacteria (Abdel-Moneim et al., 2025). These mechanisms support the role of organic acids in selectively inhibiting harmful bacteria while promoting a healthy gut microflora, thus exhibiting antimicrobial effects, thus lead to enhancing growth, feed efficiency, and better overall health in chicks.

Studying the effects of early nutrition on gene expression of specific genes involved in growth or intestinal health is essential, especially since numerous studies have shown that experimental supplements modified many gene expressions, enhancing the health and performance of chickens (Bhanja et al., 2014; Elbaz et al., 2024). Early feeding in the present experiment upregulated the relative expression of IGF-1 in the chicken liver. It is known that IGF-1 is essential for the growth and development of muscle (Fu et al., 2015). Consistent with our results, several studies have found that feeding eggs with digestible nutrients enhanced the expression of growth-related genes in chickens (Bhanja et al., 2014). Similarly, early nutrition affects the gene expression of genes responsible for muscle growth and growth hormone receptor genes, genes associated with the transport of nutrients (Peebles, 2018; Ács et al., 2025), which support muscle growth, increase growth rates, and strengthen the immune system. Additionally, there was a numerical increase in MUC2 gene expression in chickens that received the HNG than in the control group. MUC2 is a gene that encodes mucin, a protein that forms a protective layer in the intestinal lining and therefore plays a critical role in intestinal health and barrier function (Awad et al., 2025). MUC2 also provides binding sites for host bacteria and may contribute to the selection of species-specific gut microbes (Levkut et al., 2020), as well as the availability of certain nutrients to host bacteria. Similarly, a previous study reported increased MUC2 gene expression in chickens as a result of early nutrition (Elbaz et al., 2025b). The improved intestinal health associated with early nutrition may be attributed to increased MUC2 gene expression.

**In conclusion**, early feeding of ostrich chicks with hydrated nutritional gel within the first hours of hatching (2 to 6 h) has been found to enhance immune response and antioxidant defenses, improving the overall chick health. Early feeding also promotes the growth of beneficial gut microbes, which supports feed efficiency. Additionally, early feeding up-regulates IGF-1 gene expression. We conclude that early feeding has positive effects on growth performance and health in ostrich chicks. However, further research is needed to determine the need for ostriches to benefit from early feeding technology for commercial application, with a focus on nutrient metabolism and its impact on genes associated with general health traits in ostrich chicks.

## Funding

This work was supported through the Annual Funding track by the Deanship of Scientific Research, Vice Presidency for Graduate Studies and Scientific Research, King Faisal University, Saudi Arabia [Proposal Number KFU253627]. This research is also supported by 10.13039/501100004242Princess Nourah bint Abdulrahman University Researchers Supporting Project number (PNURSP2025R460), Princess Nourah bint Abdulrahman University, Riyadh, Saudi Arabia.

## CRediT authorship contribution statement

**Mohamed marzok:** Writing – review & editing, Writing – original draft, Methodology, Investigation, Funding acquisition, Data curation, Conceptualization. **Hind Althagafi:** Funding acquisition, Data curation, Methodology. **Hadeel A. Almamoory:** Data curation, Methodology. **AbdelRahman Y. Abdelhady:** Data curation. **Mohamed G. Sallam:** Data curation. **Mahmoud H.A. Mohamed:** Resources, Visualization. **Mohammed Al-Rasheed:** Resources, Visualization. **Ahmed Ateya:** Resources, Visualization, Methodology. **Salah Abdulaziz Al-Shami:** Resources, Visualization. **Ahmed I. El Sheikh:** Resources, Visualization. **Sherief M. Abdel-Raheem:** Resources, Visualization. **Moustafa Salouci:** Resources, Visualization, Data curation. **Khairiah Mubarak Alwutayd:** Resources, Visualization. **Ahmed M. Elbaz:** Conceptualization, Methodology, Data curation.

## Disclosures

The authors have declared no conflict of interest.

## Appendix Supplementary materials

Image, application 1
