# Supplementary material for: Early feeding with hydrogel nutrients modifies cecal microbiota, immunity, gene expression, and improves growth performance in ostrich chicks
Source: Poult Sci. 2025 Oct 26;104(12):106025. doi: 10.1016/j.psj.2025.106025 (PMC12648593; doi:10.1016/j.psj.2025.106025)
Supplement: Supplementary file 1 [file mmc1.pdf]

AVIBOOST AQUA-BLOK

Composition per 1 kg

Ingredients	Quantity	Ingredients	Quantity
Vitamin A	5000 iu	Sodium Salt	98 mg
Vitamin D3	750 iu	Potassium Salt	824 mg
Vitamin E	100 mg	Critic Acid	125 mg
Vitamin B1	12.5 mg	Magnesium Salt	4.6 mg
Vitamin B2	5.0 mg	Lysine Hydrochloride	10 mg
Vitamin B6	6.25 mg	Methionine	15 mg
Vitamin B12	53 µg	Essential fatty acid	5 mg
Vitamin C	10 mg	Iodine	5 mg
Biotin	12.5 µg	Selenium	1.25 mg
Niacin	14 mg	Cobalt	5 mg
Nucleotides	50 mg	Copper(MMM Chelate)	20 mg
Vitamin K3	3.5 mg	Manganese (MMM Chelate)	10 mg
Pantothenic	6.25 mg	Zinc (MMM Chelate)	30 mg
Folic Acid	6.25 mg		
